# Investigation of the Strength–Ductility Balance in an Industrial-Grade TC18 Titanium Alloy: The Pivotal Role of β Grain Size

**DOI:** 10.3390/ma19050892

**Published:** 2026-02-27

**Authors:** Jing Wang, Xiaodong Zhan, Dongdong Li, Lehua Liu, Junyang He, Jinyang Ge, Xiaoyong Zhang

**Affiliations:** 1 State Key Laboratory of Powder Metallurgy, Central South University, Changsha 410083, China; 2 Hunan Xiangtou Goldsky Titanium Industry Technology Co., Ltd., Changde 415001, China; 3 State Key Laboratory for Materials Processing and Die and Mould Technology, School of Materials Science and Engineering, Huazhong University of Science and Technology, Wuhan 430074, China; 4 National Engineering Research Center of Near-Net-Shape Forming for Metallic Materials, Guangdong Provincial Key Laboratory for Processing and Forming of Advanced Metallic Materials, South China University of Technology, Guangzhou 510640, China; liulh@scut.edu.cn; 5 School of Mechanical and Electrical Engineering, Central South University, Changsha 410083, China

**Keywords:** TC18 alloy, grain size, strain-induced martensite, work hardening rate, deformation mechanism

## Abstract

The β grain size in titanium alloys during industrial forging is critical for balancing toughness, cost-effectiveness, and processability. To address the industrial challenge of high cost and difficulty in refining β grains to the tens of micrometers scale, this study investigates the feasibility of achieving a superior strength–ductility balance in TC18 alloy with near-industrial coarse β grains (296~857 μm) under room temperature tension. A pronounced inverse correlation is observed between β grain size and both strength and ductility. The yield strength–grain size relationship follows the Hall–Petch effect, while the anomalous increase in ductility for fine-grained specimens is attributed to three factors. First, smaller grains provide a higher grain boundary density, promoting stress redistribution and mitigating stress concentrations. Second, more uniform stress distribution induces thinner, denser kink bands that enhance plasticity. Third, strain-induced martensite evolves from discrete nanoscale particles to discontinuous lines and ultimately coalesces into continuous planar bands along the (112)β and (110)β planes. This phase transformation, which initiates below a critical grain size of ~500 μm, further alleviates stress concentrations towards slip bands and contributes to dynamic work hardening. The findings demonstrate that coordinated deformation mechanisms enable excellent mechanical performance even in coarse-grained microstructures, providing a practical pathway for optimizing industrial-grade titanium alloys.

## 1. Introduction

Grain size is a fundamental microstructural parameter governing the mechanical properties of metals and alloys [1,2,3,4]. In general, a Hall–Petch formula perfectly illustrates the relationship between grain size and yield strength [5,6], which primarily arises from the interactions between grain boundaries and dislocation slip [7,8]. Such a grain size strengthening mechanism has been extensively validated in titanium alloys [9,10], demonstrating that grain refinement effectively enhances yield strength through dislocation–grain boundary interactions. On one hand, in α-titanium alloys with intrinsically limited slip systems [11,12], grain boundaries exhibit particularly strong blocking effects on dislocation motion [13,14,15]. On the other hand, in β-titanium alloys, the relationship still remains valid despite their more available slip systems and complex shearing mechanisms [16,17]. The validity of the Hall–Petch relationship in titanium alloys highlights the critical role of grain size in strength enhancement.

While the Hall–Petch relationship clearly establishes the grain size dependence of strength in titanium alloys, considering the direct impacts of grain boundaries on multiple strain carriers including dislocations and martensite [18], the correlation between grain size and overall alloy plasticity remains elusive and is still debated. For instance, Wang et al. [19] reported that Ti-4Mo-3Cr-1Fe-1Al alloys exhibited the best strength–ductility balance when the β grain size is relatively small in the range of 44−180 μm, which was attributed to the formation of martensitic band and subsequent deformation twinning (DT) and martensite reorientation during deformation. Rastogi et al. [20] found that the ductility of the Ti-10V-2Fe-3Al alloy increased with grain size from 48 μm to 106 μm. They proposed that the elastic deformation of newly transformed orthorhombic-α″, HCP-α′ martensite combined with plastic deformation of an untransformed β matrix, resulting in embrittlement. For finer β grain size, stress-induced martensite (SIM) displays a more pronounced embrittling effect. Zhu et al. [21] investigated the Ti-7333 alloy with β grain sizes ranging from 45 to 260 μm and found that plasticity initially increased and then decreased. Smaller β grains simultaneously activated stress-induced α″ martensite and multiple twinning modes, enhancing strain accommodation and work hardening, while larger β grains inhibited these transformations. In summary, studies report conflicting plasticity trends with varying grain sizes. Furthermore, the abovementioned studies primarily focus on the correlation among grain size, yield strength, and plasticity within the 100−200 μm range. In contrast, the deformation mechanisms and plasticity responses in alloys with grain sizes exceeding 200 μm have received scant attention. Elucidating these mechanisms is not only critical for fundamental understanding but also addresses a significant industrial challenge: the difficulty and high cost of controlling β grain size to the tens of micrometers scale in manufacturing processes [22].

Furthermore, research indicates that during room temperature deformation, β grain size also influences the formation of α″ martensite, primarily the content and size of the martensite phase [23]. This is attributed to the smaller β grain size altering the elastic and frictional energies required for phase transformation, thereby reducing the total resistance and lowering the martensite trigger stress [24,25]. However, the near-β titanium alloys such as TC18 retain a single β-phase after solution treatment followed by water quenching. Such alloys typically do not exhibit pronounced dual yield characteristics during deformation. Nevertheless, martensitic transformation can still occur under sustained plastic strain accumulation, a phenomenon referred to as strain-induced martensite [21,26,27,28]. Presently, the mechanism governing the formation of strain-induced martensite remains unclear, particularly across different β grain size ranges. Concurrently, the fundamental factors governing how such martensite influences the regulation of alloy strength and ductility remain to be revealed.

To address the above challenges, this study focused on an industrial-grade TC18 titanium alloy featuring coarse β-grains (297~857 μm) and systematically investigated the influence of β-grains size on the mechanical properties as well as the deformation mechanisms, including martensitic transformation and kink bands evolution. Investigating the strength and plasticity of near-β titanium alloys is crucial for optimizing their overall mechanical properties. This is because such alloys, designed with β-phase stabilizing element content slightly below the critical value, can achieve high strength through heat treatment-controlled precipitation phases while retaining the favorable plasticity of the β-phase matrix. Through quasi-in situ characterization and multi-scale organizational analysis, the dynamic formation pathway of strain-induced martensite at a specific grain size level was elucidated. Furthermore, the interacting roles of grain boundary stress coordination and martensitic synergistic deformation on work hardening behavior were revealed. This work provides a new perspective for the synergistic optimization of the strength and plasticity of near-beta titanium alloys, establishing a theoretical foundation for the industrial application of large-grained microstructures. This study helps overcome the conventional trade-off between strength and plasticity in titanium alloys, providing materials with both high specific strength and good formability for the aerospace field.

## 2. Materials and Experiments

**Figure 1:**
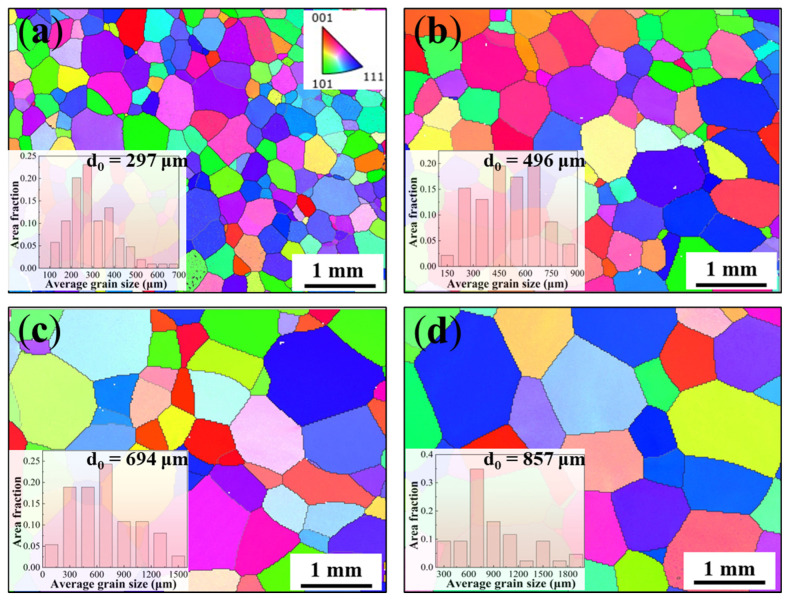
EBSD results of the as-annealed TC18 alloy after heat treatment with different solid solution temperatures and times; the inset shows the corresponding grain size distribution. (**a**) 900 °C/1 h; (**b**) 940 °C/2 h; (**c**) 980 °C/3 h; (**d**) 1020 °C/3 h.

The material used in this experiment was taken from a commercial TC18 alloy bar provided by Hunan Xiangtou Goldsky Titanium Industry Technology Co., Ltd., (Changde, China) with the chemical composition of Ti-5.16Al-4.92Mo-4.96V-1.10Cr-0.98Fe (wt.%). The ingot underwent three vacuum consumable electrode remelting processes, followed by thorough homogenization holding at 1150°C. Subsequently, it underwent two rounds of upsetting-drawing forging, with air cooling applied after forging completion. The β transfer temperature was determined as 875 ± 5 °C. Heat treatments above the β transfer temperature promoted grain growth through diffusion-driven boundary migration. Higher temperatures and longer durations increased the average β grain size, as quantified by EBSD analysis (Figure 1). To obtain different β grain sizes, heat treatments were carried out in the β-phase region at 900 °C/1 h, 940 °C/2 h, 980 °C/3 h and 1020 °C/3 h.

Dog-bone tensile specimens with a gauge dimension of 78 mm × 10 mm × 1.5 mm were extracted from the heat-treated alloy and subjected to monotonic tensile testing using an Instron 3369 mechanical testing system (Instron, Shanghai, China), with strain measured by a YYJ-4/10-L extensometer. The tensile strain rate is set as 1 × 10^−3^ s^−1^. Each test was repeated three times to ensure data reliability. Quasi-in situ tension was performed using an IBTC-500 in situ mechanical test system (CARE Measurement and Control, Tianjin, China) with a tensile displacement speed of 0.002 mm/s.

The samples for microstructure characterization were ground and mechanically polished, followed by electrochemical polishing in a solution of 10% perchloric acid, 30% n-butanol, and 60% methanol at −25 °C for 15 s. A scanning electron microscope (SEM, Helios Nano Lab G3 UC, FEI, Hillsboro, OR, USA) equipped with an electron backscatter diffraction (EBSD, Oxford Symmetry S3, Oxford Instruments, Abingdon, UK) detector and a transmission electron microscope (TEM, Talos F200X, FEI, Hillsboro, OR, USA) were used to observe the microstructure after annealing and deformation. The EBSD data processing was performed in the Orientation Imaging Microscopy (OIM) software (Version 8, EDAX, Mahwah, NJ, USA). TEM specimens were mechanically ground to a thickness of approximately 50 μm, then punched into 3 mm diameter disks. These disks were further thinned by twin jets (Denmar Struers A/S, Copenhagen, Denmark) at 20 V around −30 °C. The β grain size and thickness of the martensite and kink band were counted using the ImageJ software (Version 1.53, National Institutes of Health, Bethesda, MD, USA).

## 3. Results

### 3.1. Initial Microstructures

Figure 1 displays the inverse polar figure (IPF) and the corresponding grain size distribution of the as-annealed TC18 alloy. All four specimens exhibit fully recrystallized equiaxed β grains with random crystallographic orientations. Progressive grain growth is observed with increasing solution temperature and duration, resulting in average grain sizes of 297 μm, 496 μm, 694 μm, and 857 μm, respectively. For clarity in subsequent discussion, these samples are designated as D297, D496, D694, and D857, respectively.

### 3.2. Mechanical Properties

**Figure 2:**
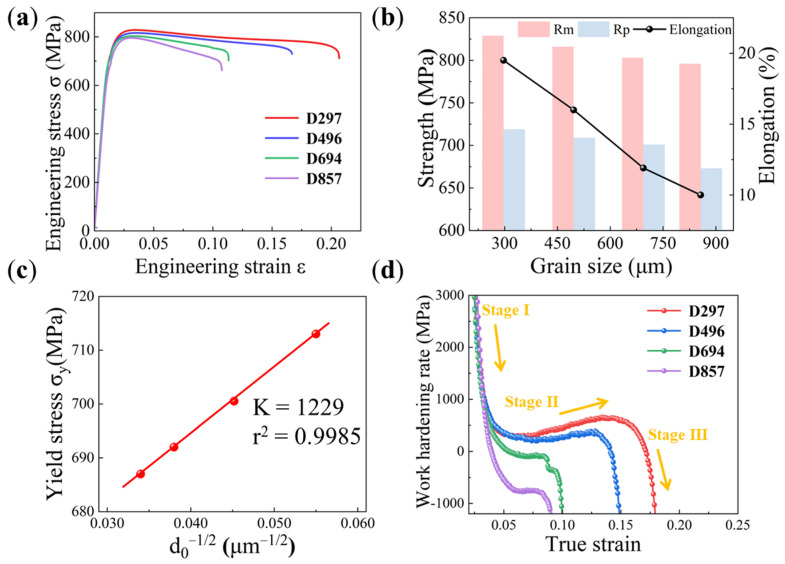
Mechanical properties of the as-annealed TC18 alloy specimens with different β grain sizes. (**a**) Engineering stress–strain curves for different β-grain sizes. (**b**) Variation in yield strength, tensile strength and elongation with β grain size. (**c**) The relationship between yield strength σ_y_ and the reciprocal of the square root of grain size d_0_^−1/2^. (**d**) Work hardening rate curves for different β-grain sizes.

The engineering stress–strain curves of the as-annealed TC18 alloy with different β grain sizes are shown in Figure 2a. As the β grain size decreases from 857 to 297 μm, the 0.2% yield strength (R_p_) increases from 719 to 829 MPa; the tensile strength (R_m_) increases from 687 to 719 MPa. Interestingly, the fracture elongation simultaneously increases, from 10% to 19.5%. (Figure 2b). The linear fitting performed on σ_y_ against d in Figure 2c demonstrates that, even with industrial-grade β grain sizes at sub-millimeter scale, the mechanical properties still exhibit a well-defined Hall–Petch relationship. The friction stress (*σ*_0_) is determined as 645.1 MPa attributed to solid solution strengthening, and the grain boundary strengthening coefficient (*k*) is measured as 1229.8 MPa·μm^1/2^. Figure 2d shows the work hardening rate profiles. Three distinct stages of work hardening are observed. The work hardening rate is determined by the slope of the tangent (i.e., d_σ_/d_ε_) on the true stress–true strain curve during the plastic deformation stage. As it is the ratio of stress increment to strain increment, its unit is the same as that of stress (i.e., MPa). In the first stage, the work hardening rate of the four specimens all decrease sharply, representing the transition from elastic to plastic deformation [29]. In the second plastic deformation stage, samples D694 and D857 exhibit typical work hardening plateaus, while D297 and D496 show a remarkable upward [30]. The third stage is characterized by a precipitous drop in work hardening rate across all specimens, indicative of significant loss of work hardening capacity. These typical three-stage features have been reported in other β-type titanium alloys Ti-4Mo-3Cr-1Fe, Ti-Mo-Zr [31,32].

### 3.3. Microstructure After Tensile Fracture

**Figure 3:**
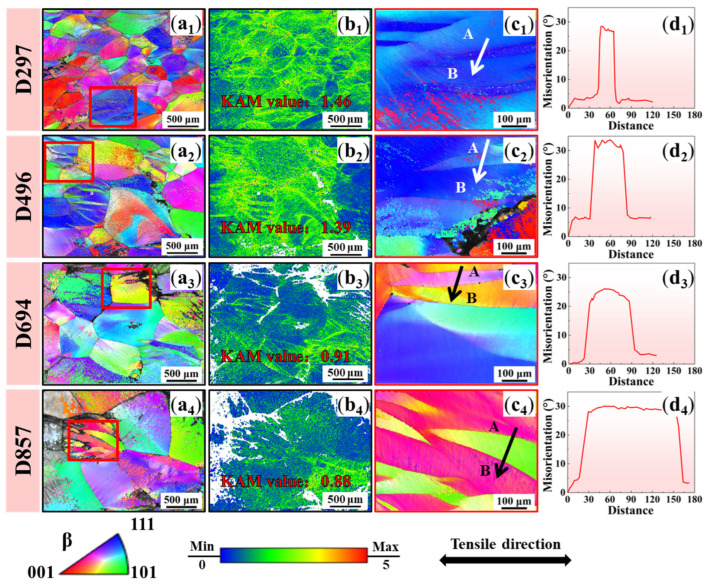
EBSD results of the as-annealed TC18 alloy after tensile fracture of specimens with different β grain sizes. (**a_1_**–**a_4_**) Low magnification inverse polar figure (IPF) map. (**b_1_**–**b_4_**) Kernel average misorientation (KAM) maps. (**c_1_**–**c_4_**) Localized enlargement of the kink bands within the red squares in figure (**a**). (**d_1_**–**d_4_**) Orientation difference along the direction of the AB arrow shown in (**c_3_**–**c_4_**).

**Figure 4:**
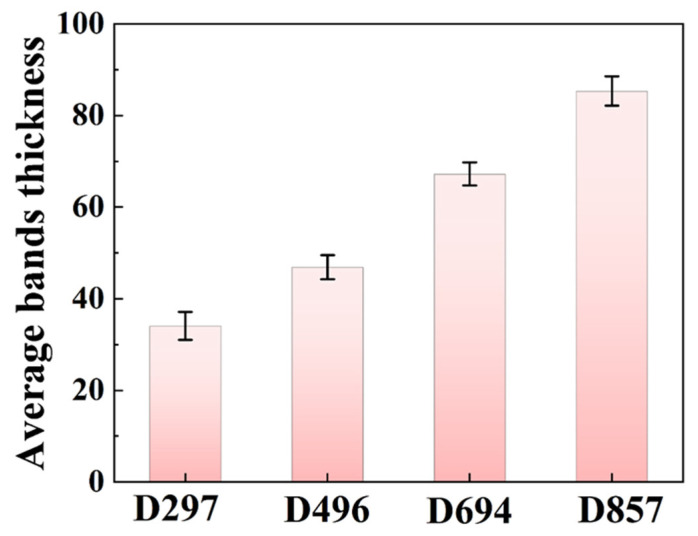
Variation in the thickness of the kink band with different β grain size. Error bars represent standard deviation (SD, *n* = 16).

Figure 3 shows the EBSD results of the as-annealed TC18 alloy with different β grain sizes after tensile fracture. The orientation within the grains exhibits variations across all four specimens (Figure 3(a_1_–a_4_)). Massive band-like structures appear within grains; see the magnified red rectangle regions in Figure 3(c_1_–c_4_). These bands clearly grow from grain boundaries but do not always terminate at them. The misorientation between the band and the surrounding β matrix is measured reaching 30° for all the four specimens, suggesting typical characteristic angles of {332}<113> twins (50.5°) and {112}<111> twins (60°) in the β-Ti [33,34]. These band-like structures are therefore termed as the kink bands [35,36]. Statistical results as shown in Figure 4, the average thickness of the kink bands shows a positive correlation with the β grain size. Moreover, significant variations in strain distributions are observed for four specimens according to the kernel average misorientation (KAM) maps (Figure 3(b_1_–b_4_)). For specimens D496 and D297, the intergranular strain distribution is relatively uniform (Figure 3(b_1_,b_2_)). The KAM values inside the kink bands are significantly reduced. For specimens D694 and D857, the intergranular and intragranular strain distributions are extremely inhomogeneous (Figure 3(b_3_,b_4_)). It can be found that the KAM values along the grain boundaries increase significantly. This indicates that with the increase in grain size, the strain distribution is more concentrated at the grain boundaries, especially at the tri-angle grain boundaries.

**Figure 5:**
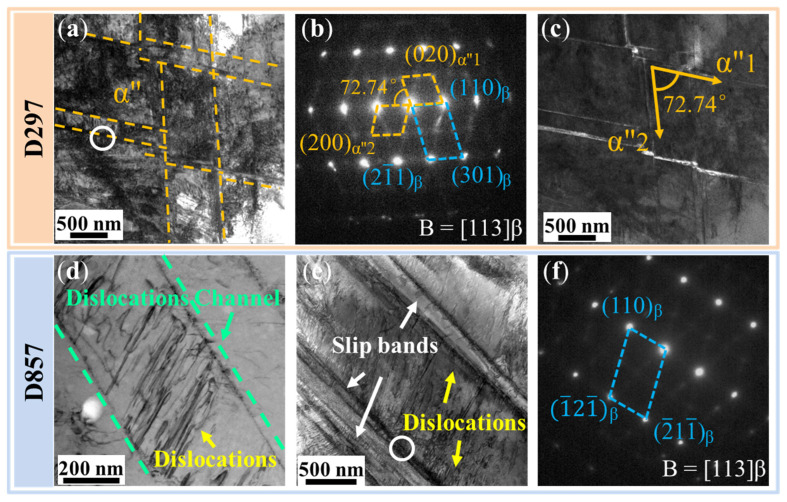
TEM results of D297 and D857 specimens after tensile fracture. (**a**) Bright-field image of D297 specimen after fracture. (**b**) SAED pattern corresponding to the white circle in (**a**). (**c**) Dark-field image of α″ martensite formation on the slip bands. (**d**) Dislocation slip channel of D857 specimen. (**e**) Bright-field image of D857 specimen after fracture. (**f**) SAED mode corresponding to the white circle in (**e**).

To further analyze the microscopic deformation mechanism of different β grain sizes, TEM characterization was performed for D297 and D857 after tensile fracture, as shown in Figure 5. For the D297 specimen, a high density of dislocations is observed in the vicinity of intersecting slip bands. According to the selected area electron diffraction (SAED) pattern and dark field image, α″ martensite is found to form within the slip bands, with a thickness ranging from 14.1 to 62.3 nm. The α″ martensite maintains an orientation relationship with the β phase as [113]_β_//[001]_α″_. The angle between α″ martensite variants in different orientations is approximately 72.74°. It is noteworthy that step-like morphologies are formed at the junctions of α″ martensite. For the D857 specimen, slip bands also develop within the β grains, functioning as channels for dislocation slip (Figure 5d). With increasing deformation, dislocations within these bands gradually accumulate and tangle, forming the regions with high dislocation density (Figure 5f). However, α″ martensite is absent within these slip bands (Figure 5e), indicating that an increase in β grain size hinders the formation of α″ within the grains.

### 3.4. Microstructure Evolution During Tension

**Figure 6:**
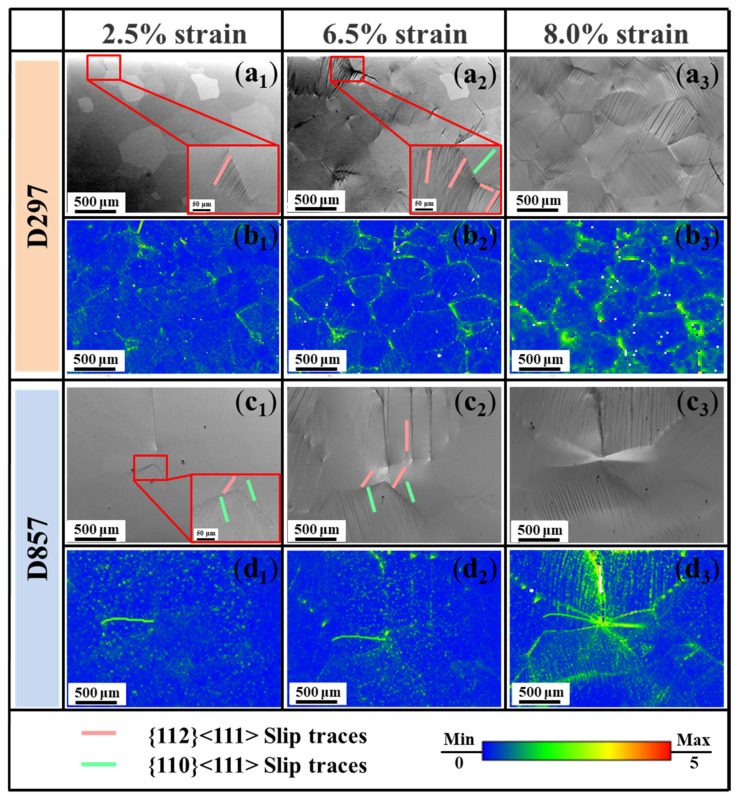
Quasi-in situ EBSD results of D297 and D857 specimens at different strains. (**a_1_**–**a_3_**) and (**c_1_**–**c_3_**) are the band contrast maps of samples D297 and D857 at strains of 2.5%, 6.5%, and 8.0%, respectively. The red square indicates the local magnification of the slip bands. (**b_1_**–**b_3_**) and (**d_1_**–**d_3_**) are the KAM maps of D297 and D857 at strains of 2.5%, 6.5%, and 8.0%, respectively.

Figure 6 demonstrates the quasi-in situ EBSD results of D297 and D857 specimens. At 2.5% tensile strain, parallel slip traces are observed hitting grain boundaries in both D297 and D857 specimens. Slip trace analysis indicates the activated slip systems as {110}<111>, {112}<111> (Figure 6(a_1_,c_1_)), in line with the reported β-Ti alloys. The corresponding kernel average misorientation (KAM, Figure 6(b_1_–b_3_,d_1_–d_3_)) maps indicate that most internal strain accumulates in the vicinity of the grain boundaries within the early deformation stage. When the tensile strain increases to 6.5%, the number of slip traces increases and slip steps become more significant. The slip traces with different orientations emerged within β grains. At the same time, the concentrated strain towards grain boundaries further intensifies. Upon further straining to 8.0%, in Figure 6(a_3_), slip traces develop across the entire β grain interiors in the D297 specimen, while the corresponding KAM map (Figure 6(b_3_)) suggests that the intergranular strain level simultaneously increases. When compared to the D857 specimen where microcracks are triggered along the overstressed grain boundaries (Figure 6(c_3_)), the increased grain boundary strain level in the D297 specimen is somehow limited, and the strained regime near the grain boundary also seems enlarged. This suggests that with decreasing grain size, stress is more readily accommodated by the increased density of grain boundaries, resulting in a more uniform stress distribution.

**Figure 7:**
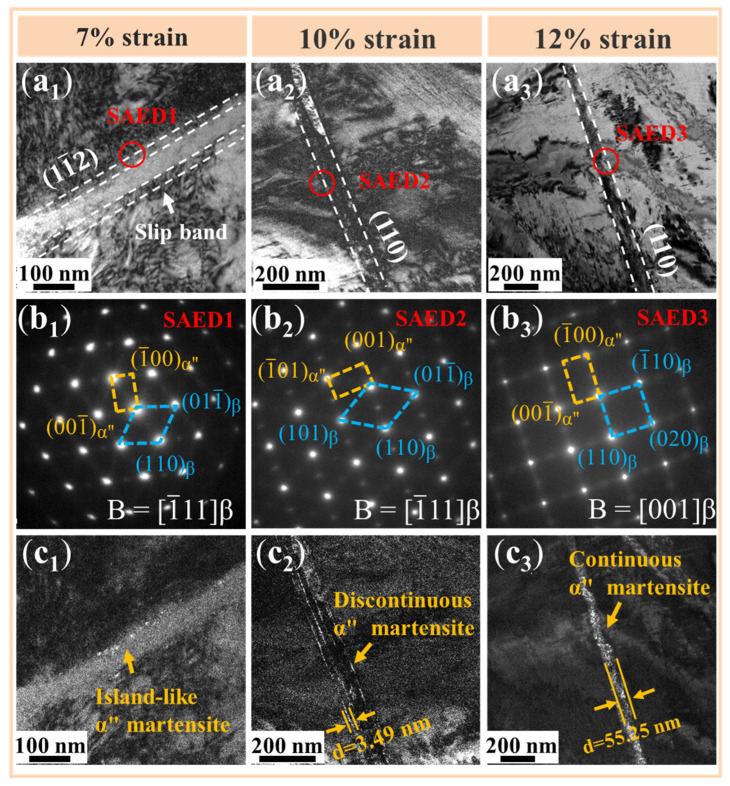
TEM results of the slip band in relation to α″ martensite after tensile fracture of D297 specimen. (**a_1_**–**a_3_**) Bright-field image of the slip band organization. (**b_1_**–**b_3_**) SAED pattern corresponding to the red circle in (**a_1_**–**a_3_**), taken from the interface between the slip band and the matrix. (**c_1_**–**c_3_**) Dark-field image of α″ martensite formation in the slip band in panel (**a_1_**–**a_3_**).

To further clarify the nucleation and progression of α″ martensite, TEM characterization of D297 specimens at different overall strain levels was carried out; see Figure 7. When the tensile strain is 7%, the α″ martensite nucleates and grows discretely along the β slip band boundary, presenting island-like characteristics (Figure 7(c_1_)). The direction of this slip band is {1$\bar{1}$2}_β_. With the tensile strain increasing to 10%, the above α″ martensite first lengthens into discontinuous bands with a thickness of 3.49 nm, gradually covering the slip band boundary (Figure 7(c_2_)). The direction of this slip band is {110}_β_. With the tensile strain further rising to 12%, these α″ martensite bands further connect and then apparently thicken, with an average thickness of 55.25 nm. (Figure 7(c_3_)). And the direction of the slip band is still {110}_β_. In summary, slip bands typically form along the close-packed {110} and {112} planes in bcc structures. This indicates that plastic deformation primarily initiates on these slip planes, which leads to local lattice distortion and energy accumulation. This results in high strain level in the slip bands, making it a favorable region for martensitic phase transformation.

## 4. Discussion

### 4.1. The Martensitic Transformation: β Grain Size Scale Effect

In the near or meta β titanium alloys, the β → α″ martensitic phase transformation typically serves as a pivotal mechanism in modulating their strength and plasticity [37,38]. Here, we first consider the types of the phase transformation: stress-induced and strain-induced. Stress-induced martensitic transformation occurs when the shear stress directly acts on the bcc lattice of the β phase, enabling the alloy to overcome the transformation energy barrier through lattice shear [39]. Typically, the stress–strain curves exhibit a double-yield feature [40,41]. Nevertheless, when the critical stress-inducing martensitic transformation is higher than the yield strength of the parent phase, the parent phase undergoes plastic strain. At this point, it is referred to as strain-induced martensitic transformation. The occurrence of strain-induced martensite depends on the accumulated strain, requiring a critical plastic strain to initiate the phase transformation. There is no obvious stress plateau in the stress–strain curve, and the work hardening curve exhibits progressive hardening. This phase transformation is generally irreversible [42]. In this study, the tensile stress–strain curves of all four samples exhibited no double-yield feature. Furthermore, martensitic nucleation initiates in the D297 specimen at 7% strain, forming island-like distributions within the slip band (Figure 7(c_1_)). And as the strain increases, the martensite gradually coarsens. Therefore, it can be considered that the martensitic phase transformation in this study is strain-induced.

Next, we dig into the roles played by grain size on martensitic phase transformation behavior. Thermodynamically, the onset of martensitic transformation in β titanium alloys in general requires the following, as shown in Equation (1) [43]:

(1) $$∆G_{t}=∆G_{c}+∆G_{nc}\leq 0$$

where Δ*G_t_*, Δ*G_c_*, Δ*G_nc_* are the total, chemical, and non-chemical driving forces, respectively. Δ*G_c_* represents the Gibbs free energy difference between the β and α″ phases. The non-chemical driving forces Δ*G_nc_* include the shear strain energy (Δ*G_sh_*), the elastic strain energy (Δ*G_el_*), the dislocation storage energy (Δ*G_st_*) and the interfacial energy Δ*G_in_*, making the following, as shown in Equation (2):

(2) $$∆G_{nc}=∆G_{sh}+∆G_{el}+∆G_{st}+∆G_{in}$$

Based on the literature [43,44,45], each term in the right part of Equation (2) can be further expressed as follows, as shown in Equations (3)–(6):

(3) $$∆G_{sh}=\frac{b}{d}\tau V_{m}$$

(4) $$∆G_{el}=\frac{1}{2\rho }\left(\frac{\sigma^{2}}{E_{M}}-\frac{\sigma^{2}}{E_{\beta }}\right)$$

(5) $$∆G_{st}=\rho \mu b^{2}V_{m}$$

(6) $$∆G_{in}=\frac{2\sigma V_{m}}{r}$$

where *b* is the Burgers vector, *d* is the interatomic spacing of the shear plane, *τ* is the shear stress and *V_m_* is the total deformed molar volume; *E_M_* and *E_β_* are the Young’s moduli of the martensite and β phases, respectively, *ρ* is dislocation density and *μ* is shear modulus, and *r* is the effective curvature radius of the martensite plate/interface. *σ* is the interfacial energy per unit area between the martensite and the β matrix. Finally, the total driving force of martensitic transformation becomes the following, as shown in Equation (7):

(7) $$∆G_{t}=∆G_{c}+\frac{b}{d}\tau V_{m}+\frac{1}{2\rho }\left(\frac{\sigma^{2}}{E_{M}}-\frac{\sigma^{2}}{E_{\beta }}\right)-\rho \mu b^{2}V_{m}+\frac{2\sigma V_{m}}{r}$$

From Equation (7) above, Δ*G_c_*, Δ*G_in_*, and Δ*G_el_* are all independent of grain size [45]. Δ*G_sh_* directly overcomes the transformation energy barrier during the initial deformation stage (elastic regime) and becomes the dominant factor for stress-induced martensite formation. Through dislocation accumulation during plastic deformation, Δ*G_st_* provides the primary driving force for strain-induced martensitic transformation. In this item, *ρ* is related to the β grain size and can be expressed as *ρ* = *ρ*_0_ *+ kε*/*d*_0_; *d*_0_ is the β grain size and ε is the strain. When the β grain size is reduced, the dislocation energy storage capacity increases significantly. In the case of the fractured D297 specimen, the measured KAM value reaches 1.46 (Figure 3(b_1_)), which effectively promotes the martensitic phase transformation. Larger β grains demand higher strains to reach similar dislocation densities. However, specimens fracture before reaching these critical strain levels during deformation, which fundamentally explains the suppression of martensite formation in specimens with coarser β grains.

**Figure 8:**
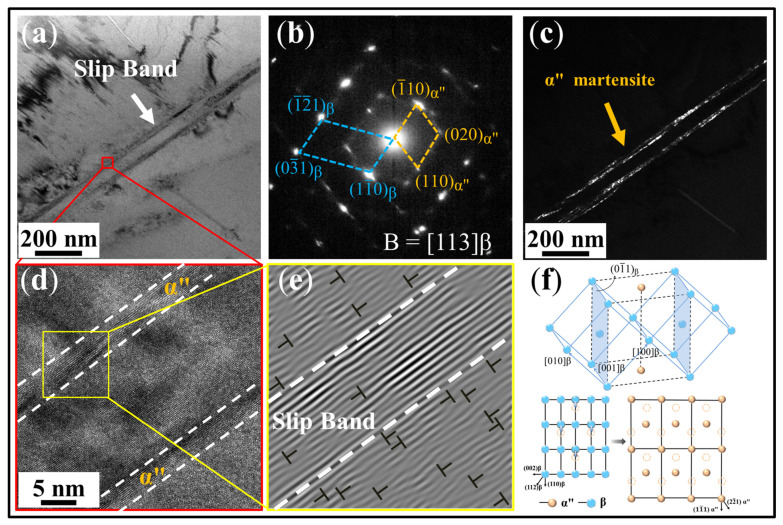
TEM results of D297 specimen forming martensite at 10% strain. (**a**) Bright-field image of the slip bands. (**b**) SAED pattern corresponding to figure (**a**). (**c**) Dark-field image of the formation of martensite in the slip bands. (**d**) HRTEM image of the slip bands corresponding to Figure (**a**). (**e**) IFFT image of the dislocation distribution of the slip band and the matrix, and (**f**) dot-matrix correspondence between the β and α″ phases.

To confirm role of martensitic transformation in dissipating stored dislocation energy, TEM characterization was performed on the 10% strained D297 sample, as shown in Figure 8. During deformation, two parallel slip bands form, surrounded by dislocation tangles (Figure 8a). α″ martensite nucleates and elongates along these bands (Figure 8c). Analysis by high-resolution transmission electron microscopy (HRTEM) reveals distinct atomic arrangements between the α″ martensite and β matrix (Figure 8d). IFFT (Inverse Fast Fourier Transform) imaging demonstrates the high density of edge dislocations at the β matrix/band interface, while the dislocation density within the bands is markedly reduced (Figure 8e), suggesting that α″ martensite formation alleviates dislocation accumulation. This indicates that dislocation pile-up during deformation induces local strain concentration within the bands, providing the driving force for α″ martensite nucleation. This transformation follows a shear-dominated mechanism. Similarly, Yao et al. [46] observed the β → α″ transformation in Ti2448 alloy during tensile deformation, revealing nanoscale lamellar α″ within slip bands. The phase transformation corresponds to a contraction of the [001]_β_ axis, an expansion of the [$\bar{1}$10]_β_ axis, and a shuffle of atoms along the <011>_β_ direction (Figure 8f) [47,48,49].

### 4.2. Plasticity Enhancement by β Grain Size Refinement

Clearly, the effect of grain size on strength for the TC18 alloy follows the classical Hall–Petch relationship [50,51]. Conversely, for the overall alloy plasticity, we detect a similar negative correlation with the β grain size. The same is for the work hardening capability. The underlying mechanism of these observations can be interpreted from three distinct aspects.

Firstly, the overall strain distribution. Grain boundaries serve as obstacles to dislocation motion, with their intrinsic stress concentration originating from impeded dislocation transmission across boundaries, which consequently leads to localized strain energy accumulation [50]. Notably, in fine-grained materials, this inherent characteristic can be effectively harnessed as a pivotal mechanism for enhancing plastic deformability. In the present study, the uniformly distributed high-density grain boundaries in specimen D297 facilitate effective spatial stress redistribution. Specifically, the stress is no longer concentrated at individual grain boundaries but rather achieves coordinated multiscale distribution among adjacent grains (Figure 6(b_3_)). This strain partitioning mechanism effectively mitigates localized strain concentration and significantly elevates the critical condition for microcrack nucleation. Simultaneously, the enhanced grain boundary–dislocation interactions contribute to improved work hardening capability. In contrast, the D857 specimen that is characterized by fewer grain boundaries demonstrates limited stress coordination capability. This leads to significant stress concentration at the triple crystal boundary (Figure 6(d_3_)), ultimately initiating microcrack formation and premature fracture.

Secondly, the formation of kink bands. These are characterized as localized slip bands exhibiting arbitrary crystal rotation within grains [52]. Kink band proliferation forms adaptive shear channels that mitigate stress localization via self-organized geometric softening. In this study, β grain size is negatively correlated with kink band density and positively correlated with kink band thickness. For sample D297, high-density kink bands effectively distribute strain to multiple microbands, mitigating strain localization at individual slip bands or grain boundaries. Kink bands dynamically adjust their orientations during deformation, further mitigating strain incompatibility. Moreover, the high density of twisted band boundaries serves as a potent barrier to dislocation motion, promoting dislocation accumulation. This mechanism substantially enhances the work hardening rate [53,54].

Thirdly, the strain-induced martensitic transformation. As discussed in Section 4.1, martensite formation consumes dislocation energy storage, effectively alleviating local stress concentration caused by dislocation accumulation and consequently enhancing plastic deformation capacity. For the D297 sample, deformation induces the formation of dense dislocation tangles surrounding slip bands. The subsequent martensitic transformation effectively consumes the strain energy stored in these slip bands. In addition, when multiple martensite variants nucleate and intersect during deformation, the martensite that forms first obstructs the growth path of subsequent variants, forcing new variants to adjust their growth path or fracture. This intersection process requires martensite boundaries to overcome frictional resistance, thereby consuming substantial mechanical energy (Figure 5c). The energy dissipation associated with the martensitic transformation effectively mitigates localized stress concentration, leading to markedly improved plastic deformability. And the evolution of α″ martensite from discrete islands to interconnected bands significantly influences the mechanical behavior of the material. The initial nucleation and gradual coalescence of martensite contribute to continuous strain hardening by introducing additional barriers to dislocation motion, thereby enhancing the work hardening rate [55]. Meanwhile, the transition to a continuous martensitic network improves uniform elongation by accommodating plastic strain more effectively.

**Figure 9:**
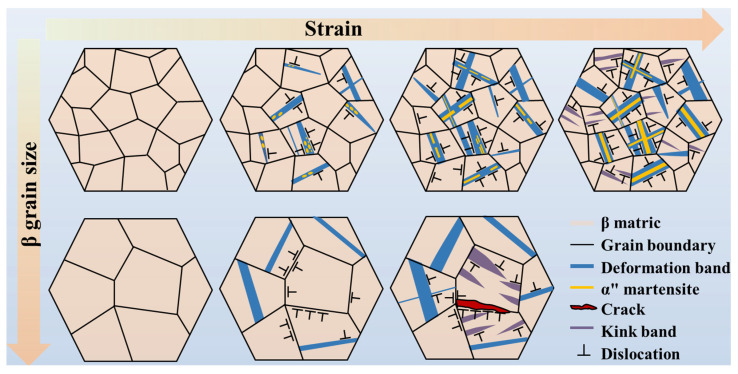
Schematic diagram of microstructure evolution with changes in β grain size and tensile strain.

In conclusion, the deformation mechanisms of TC18 alloy with the industrial-grade grain sizes are schematically illustrated in Figure 9. Upon reaching the critical stress for strain-induced martensitic α″ transformation, the α″ martensite nucleates preferentially at the pre-existing slip bands, initially manifesting as discrete island-like precipitates. With progressive strain accumulation, the martensite lengthens into discontinuous bands, ultimately developing into continuous bands at higher strain levels. During the final deformation stages, continued thickening of martensite bands occurs concurrently with the formation of kink bands, which collectively contribute to strain accommodation. The deformation behavior in the fine-grained specimens is thus governed by the synergistic interplay between dislocation slip, strain-induced martensitic transformation, and kink band formation. In contrast, the coarse-grained specimens exhibit different deformation mechanisms, in which martensitic transformation is suppressed. The deformation process in such specimens is primarily mediated by dislocation slip and kink band formation. It should be noted that while β grain elongation occurs during plastic deformation to accommodate applied strains, this geometric change has been omitted from the schematic representation for clarity.

## 5. Conclusions

In this work, the effect of near-industry grain size on mechanical properties as well as the deformation mechanism for TC18 alloy during room temperature tensile deformation were investigated. The main conclusions are as follows.

1. The relationship between yield strength and the β grain size conforms to the Hall–Petch effect. As the grain size decreases, plasticity improves from 10% to 19.5%, and the work hardening rate is also considerably elevated.
2. Dislocation slip and kink bands dominate the mechanical response across four grain sizes for TC18 alloy during the tensile process. As the β grain size decreases, the kink bands become progressively finer and more densely distributed. Moreover, the martensite transformation is found within the finer grains. And the martensite shifts from island-like to band-like structures as the strain increases.
3. The formation of martensite is primarily attributed to dislocation energy storage during the tensile process, distinct from typical stress-induced martensite. Specifically, the martensite nucleates and grows primarily along the {112}<111> and {110}<111> slip bands, owing to the high stress concentration and lattice distortion present in these regions.
4. The enhanced plasticity with grain refinement arises from three synergistic mechanisms: reduced stress concentration at grain boundaries, adaptive stress redistribution through kink band networks, and martensitic transformation that buffers localized stress on slip bands, thereby promoting uniform deformation and elevating work hardening.

## Data Availability

The original contributions presented in this study are included in the article. Further inquiries can be directed to the corresponding author.
